# Semantic Processing in Autism Spectrum Disorders Is Associated With the Timing of Language Acquisition: A Magnetoencephalographic Study

**DOI:** 10.3389/fnhum.2020.00267

**Published:** 2020-07-10

**Authors:** Banu Ahtam, Sven Braeutigam, Anthony Bailey

**Affiliations:** ^1^Fetal-Neonatal Neuroimaging and Developmental Science Center, Department of Pediatrics, Division of Newborn Medicine, Boston Children’s Hospital and Harvard Medical School, Boston, MA, United States; ^2^Oxford Centre for Human Brain Activity, Wellcome Centre for Integrative Neuroimaging, Department of Psychiatry, University of Oxford, Oxford, United Kingdom; ^3^Department of Psychiatry, Faculty of Medicine, University of British Columbia, Vancouver, BC, Canada

**Keywords:** autism, language, magnetoencephalography, N400, semantics

## Abstract

Individuals with autism show difficulties in using sentence context to identify the correct meaning of ambiguous words, such as homonyms. In this study, the brain basis of sentence context effects on word understanding during reading was examined in autism spectrum disorder (ASD) and typical development (TD) using magnetoencephalography. The correlates of a history of developmental language delay in ASD were also investigated. Event related field responses at early (150 ms after the onset of a final word) and N400 latencies are reported for three different types of sentence final words: dominant homonyms, subordinate homonyms, and unambiguous words. Clear evidence for semantic access was found at both early and conventional N400 latencies in both TD participants and individuals with ASD with no history of language delay. By contrast, modulation of evoked activity related to semantic access was weak and not significant at early latencies in individuals with ASD with a history of language delay. The reduced sensitivity to semantic context in individuals with ASD and language delay was accompanied by strong right hemisphere lateralization at early and N400 latencies; such strong activity was not observed in TD individuals and individuals with ASD without a history of language delay at either latency. These results provide new evidence and support for differential neural mechanisms underlying semantic processing in ASD, and indicate that delayed language acquisition in ASD is associated with different lateralization and processing of language.

## Introduction

Autism spectrum disorders (ASD) are associated with impairments in reciprocal social interaction and communication, as well as stereotyped, restricted, and repetitive patterns of interests and behaviors (American Psychiatric Association, 2013). Individuals with ASD can show varying impairments in semantics, syntax, phonology, morphology, reading, and narrative ability, in addition to impaired social language usage (Tager Flusberg et al., 2005; Prelock and Nelson, 2012). Regardless of language ability, individuals with ASD tend to interpret speech literally, not taking context into account (Happé, 1997; Jolliffe and Baron-Cohen, 1999). Affected individuals are also less likely to use sentence context spontaneously to correctly pronounce homographs, showing a tendency to pronounce the dominant meaning (DH) of a homograph regardless of sentence context (Frith and Snowling, 1983; Snowling and Frith, 1986). Poor performance on the pronunciation of the rare meaning of homographs is independent of sentence position (Happé, 1997; Jolliffe and Baron-Cohen, 1999; Lopez and Leekam, 2003; Brock et al., 2017). The impairments on pronunciation tasks suggest that contextually inappropriate understanding of speech is not attributable entirely to deficits in social cognition. Indeed, it has been suggested that the finding relates to a broader weakness in processing information in context (Jolliffe and Baron-Cohen, 1999), sometimes referred to as the “Weak Central Coherence” theory, which hypothesizes that individuals with autism have difficulties in integrating pieces of information to establish a whole meaning, showing a bias toward local rather than global processing (Frith, 1989).

Investigation of the neural mechanisms underlying semantic processing anomalies in ASD has largely focused on the N400 response and most studies have neglected early event related potential (ERP) and event related field (ERF) responses. The amplitude of the N400 is greater for words that violate semantic context or that are less likely to occur in a sentence context although semantically legitimate (Kutas and Hillyard, 1980, 1984a, 1984b; Kutas et al., 1984; Kutas and Federmeier, 2000), and is smaller for highly predictable words (Maess et al., 2016). N400 studies comparing ASD and typically developing (TD) individuals have found reduced amplitude (Pijnacker et al., 2010) suggesting difficulties in integrating words rapidly to establish meaningful context (Bailey et al., 2000; Pijnacker et al., 2010), or the use of different neuronal networks in semantic processing in ASD (Braeutigam et al., 2008; McCleery et al., 2010).

Most language processing studies of ASD have neglected early ERP/ERF responses (for an exception see Braeutigam et al., 2008). Early ERP/ERF responses were initially considered to index the processing of surface features of written words (Solomyak and Marantz, 2009); however, some EEG and MEG studies have shown that semantic information is accessed as early as 100–200 ms after visual word onset (Moscoso del Prado Martin et al., 2006; Penolazzi et al., 2007; Shtyrov and Pulvermuller, 2007). Semantic expectedness of a word in sentence context (Sereno et al., 2003; Penolazzi et al., 2007) and semantic ambiguity have been shown to affect ERP/ERFs as early as 100–200 ms (Federmeier et al., 2000) after stimulus onset. Differences between the early ERPs to congruous and incongruous words have also been found (Penolazzi et al., 2007; Shtyrov and Pulvermuller, 2007), suggesting that semantic context might influence processing within 200 ms of the onset of a written word (Penolazzi et al., 2007).

It is now generally accepted that many language skills and processes occur in a complex network of cortical and subcortical circuits distributed across the brain (Démonet et al., 2005; Fisher and Marcus, 2006; Kujala et al., 2007). Although the left hemisphere is dominant for most language functions (e.g., syntactic and phonetic processing, deciphering the meaning of words and sentences), the right hemisphere also plays some role in areas such as comprehension, pragmatic aspects of language, and coherent discourse (Kaplan et al., 1990; Carroll, 2007; Papadatou-Pastou, 2011). Functional MRI studies find that about 95% of typically developing and healthy right-handed individuals demonstrate left hemispheric activity for language processing (Pujol et al., 1999; Springer et al., 1999; Knecht et al., 2000). Furthermore, among healthy non-right-handed individuals, approximately 78% of the adults (Szaflarski et al., 2002) and 85% of the children (Szaflarski et al., 2012) show left-hemispheric lateralization for language. In an fMRI study, sentence reading was shown to especially engage the following areas of the left hemisphere: inferior frontal gyrus, angular gyrus, supramarginal gyrus, fusiform gyrus, superior temporal gyrus (Constable et al., 2004). Stowe et al. (2004) used PET to study sentence comprehension when subjects read ambiguous and unambiguous sentences. They found that left inferior frontal gyrus, the right basal ganglia, the right posterior dorsal cerebellum, and the left median superior frontal gyrus were activated more during ambiguous sentence reading. Ambiguities in sentence contexts were also shown to increase activation in the left inferior temporal/fusiform gyrus (Kuperberg et al., 2000) and posterior left superior temporal gyrus (Friederici, 2003). Based on their MEG experiment results, Kujala et al. (2007) suggested that a left hemisphere neuronal network underlay reading comprehension. This network was similar for isolated words and connected text, and did not change according to word presentation speed.

Functional and electrophysiological studies of language processing in ASD have found an atypical pattern of hemispheric specialization, with reduced activity in the left temporal language regions and/or increased activity in the right temporal language regions compared with TD individuals (Flagg et al., 2005; Braeutigam et al., 2008; Knaus et al., 2010; Pijnacker et al., 2010; Eyler et al., 2012). Such unusual patterns of hemispheric activity in ASD are seen across almost the entire breadth of language processing, including processing of congruous and incongruous sentence final words (Braeutigam et al., 2001, 2008). Structural MRI and diffusion studies have also found reduced or reversed hemispheric asymmetry in language-related regions in individuals with ASD (Nakada et al., 2001; Rojas et al., 2002, 2005; Koldewyn et al., 2014; Verly et al., 2014). Despite apparently unusual lateralization of language, the relationship with other mechanisms and the development of language are unclear. Although the prevalence of left-handedness in ASD is high (Markou et al., 2017), greater right hemispheric activity in ASD does not show a systematic relationship with left-handedness (Dawson et al., 1986; Braeutigam et al., 2008; Knaus et al., 2010). Additionally, previous research has shown that individuals who had delayed language acquisition, due to congenital deafness and being born to hearing parents, had a higher prevalence of non-right-handedness and atypical hemispheric lateralization. It has been suggested that, in these individuals, delayed access to linguistic input might affect lateralization processes in the brain (Papadatou-Pastou and Sáfár, 2016).

The question of whether delayed language onset in ASD matters has been long debated (Howlin, 2003; Lai et al., 2014), but no clear conclusions have been reached, in part due to variations in age as well as level of intellectual and language abilities of participants in the various studies (Macintosh and Dissanayake, 2004; Witwer and Lecavalier, 2008; Tsai, 2013). Several groups have investigated the neuroanatomical correlates of early language development in ASD and found differences in the gray and white matter volumes between LD and NLD groups (Kwon et al., 2004; Lotspeich et al., 2004; McAlonan et al., 2008, 2009; Toal et al., 2010; Lai et al., 2014; Floris et al., 2016). Recent EEG studies showed differences between the ASD sub-groups, indicating distinct neurophysiological characteristics between individuals with and without a delayed language onset (Pijnacker et al., 2010; Duffy et al., 2013). Moreover, transcranial magnetic stimulation (TMS) studies have also found that ASD individuals with a history of language delay show less cortical inhibition in the left hemisphere compared to those with no language delay (Enticott et al., 2010, 2013). Additionally a significant difference between the NLD and LD groups was observed in the association between cortical thickness and verbal IQ in the parieto-occipital regions (Balardin et al., 2015). Lai et al. (2014) found structural brain differences between adult males with ASD with and without language onset delay.

The main goal of this magnetoencephalographic (MEG) study of reading was to investigate sentence context effects on the understanding of individual words and semantic ambiguity resolution in ASD and TD individuals. As an ancillary analysis we also studied the relationship between the history of language acquisition and language processing in ASD individuals to examine the potential importance of language development versus current language ability. We predicted that individuals with ASD would show reduced sensitivity to sentence context reflected in reduced amplitude of responses compared to TD individuals, and that semantic ambiguity resolution in ASD would show different underlying mechanisms, based on our previous work (Bailey et al., 2000; Ahtam, 2009) shown by increased right hemispheric activity at the N400 latencies, compared to TD. We also expected to find differences in brain activity between the ASD subgroups with and without a history of delayed language onset, where the ASD group with delayed language onset was expected to show non-significant responses to different final word categories at early latencies, and strong right hemispheric activity at the N400 latency. The analyses were conducted in the sensor space because insufficient anatomical MRI scans were available.

## Materials and Methods

### Participants

Participants with ASD were recruited from Oxfordshire and other southern counties in England through the National Autistic Society, local ASD parent support groups, schools, residential units, and colleges for individuals with ASD. Posters and leaflets were displayed in schools, sports and community centers within Oxfordshire to recruit TD participants. Fifty individuals participated in the experiment, but for technical reasons (e.g., problems with head-position measurements or triggers) the data from six individuals (three individuals from each participant group, TD and ASD) had to be excluded; consequently data were analyzed from 44 participants. Refer to Table 1 for the descriptive statistics of the study cohorts. In the ASD group (*n* = 22) there were twenty males and two females; in the TD group (*n* = 22) there were nineteen males and three females. The mean age in both TD and ASD groups was 20 years (Age ranges: TD = 15–33 years; ASD = 14–43 years (the second oldest individual in the ASD group was 33 years old). The clinical diagnosis of an ASD was confirmed using the Autism Diagnostic Interview-Revised (ADI-R) (Le Couteur et al., 2003) and the Autism Diagnostic Observation Schedule (ADOS) (Lord et al., 1999). All TD participants were reported to have normal language development, which was confirmed with the results of the following psychometric tests conducted with all participants: Wechsler Abbreviated Scale of Intelligence (WASI) (Wechsler, 1999); word reading, pseudoword reading, and reading comprehension measured by the Wechsler Individual Achievement Test (WIAT-II) (Wechsler, 2005), the Test for Reception of Grammar-Electronic (TROG-E) (Bishop, 2005), and the British Picture Vocabulary Scale (BPVS-II) (Dunn et al., 1997). In the ASD group, language onset was characterized using ADI-R questions 9 and 10, which record the ages in months of first single words and first phrases (First words cannot be “mama” or “dada” and had to be used consistently by the child with meaning on more than one occasion and with the purpose of communication; first phrases had to consist of at least two words, one of which had to be a verb and could not have been learnt as a single unit). The ADI-R defines delayed language onset as the age of first single words equal to or later than 24 months and/or age of first phrases equal to or later than 33 months. Individuals were considered to have delayed language onset, if the age of first single words was equal to or later than 24 months or if the age of first phrases was equal to or later than 33 months. In all but one ASD individual, there was either no history of language delay or the individual was delayed on both measures. The one exception acquired first words at 18 months and phrases at 42 months and was allocated to the language delay group. Of the 22 ASD participants, 12 had no history of delayed language acquisition (NLD) and 10 had a history of language delay (LD). In the NLD group, mean age of first single words was 14.5 (±4.1) months and the mean age of first phrases was 21.2 (±4.4) months; in the LD group the ages were 36.4 (±14.5) and 54.4 (±16.1) months respectively. According to the results of independent *t*-tests, the LD group had significantly delayed language onset compared to the NLD group both for first single words [*t*(19) = −5.33, *p* < 0.001, *r* = 0.77] and first phrases [*t*(19) = −5.88, *p* < 0.001, *r* = 0.8]. Handedness was assessed via self-report. All participants reported clear handedness and none were ambidextrous. In both the TD and ASD participant groups there were 6 left-handed individuals (one left-handed female in each group); five (all male) of the six left-handed ASD individuals had a history of language delay. Participants were native speakers of British English. All participants had normal or corrected-to-normal vision. According to the report by their parents/caregivers or self-report, no TD or ASD participants had any neurological disorder and none of the participants were taking any central nervous system (CNS) medication.

**TABLE 1:** Pre-assessment results for individuals who participated in the study.

	Participant Group	Mean	*SD*	Min.	Max.	*N*	*P*-value
Age	TD	20.00	5.01	15	33	22	0.903
	ASD	20.23	7.06	14	43	22	
	No-delay	17.92	4.21	14	28	12	0.055
	Delay	23.00	8.88	15	43	10	
Verbal IQ (WASI)	TD	112.95	11.45	92	140	22	0.521
	ASD	110.05	17.64	70	133	22	
	No-delay	114.17	15.90	80	133	12	0.224
	Delay	105.10	19.17	70	130	10	
Performance IQ (WASI)	TD	110.05	10.42	89	127	22	0.152
	ASD	106.24	13.01	83	128	21	
	No-delay	104.83	15.10	83	128	12	0.780
	Delay	108.11	10.13	93	123	9	
Full IQ (WASI)	TD	112.77	9.13	92	138	22	0.195
	ASD	108.90	14.72	86	133	21	
	No-delay	110.42	14.85	86	133	12	0.357
	Delay	106.89	15.18	86	129	9	
Receptive Vocabulary (BPVS)	TD	147.36	9.78	116	163	22	0.017*
	ASD	134.82	21.20	87	161	22	
	No-delay	136.50	24.87	87	161	12	0.588
	Delay	132.80	16.86	107	154	10	
Receptive Grammar (TROG-E)	TD	85.32	11.34	67	104	22	0.163
	ASD	90.50	12.84	55	109	22	
	No-delay	92.33	11.96	67	109	12	0.481
	Delay	88.30	14.14	55	104	10	
Word reading list (WIAT)	TD	128.32	2.82	118	131	22	0.046*
	ASD	126.00	4.43	117	131	22	
	No-delay	125.33	4.58	117	131	12	0.636
	Delay	126.80	4.34	118	131	10	
Pseudoword Reading list (WIAT)	TD	50.82	2.28	46	54	22	0.102
	ASD	49.27	3.67	40	53	22	
	No-delay	48.83	3.59	42	53	12	0.771
	Delay	49.80	3.88	40	53	10	
Reading Comprehension (WIAT)	TD	46.86	5.44	32	57	22	0.325
	ASD	44.68	8.69	26	55	22	
	No-delay	46.83	7.98	26	55	12	0.088
	Delay	42.10	9.21	28	55	10	
Reading Speed (WIAT)	TD	326.45	69.78	198	557	22	0.717
	ASD	337.90	126.40	153	644	21	
	No-delay	321.75	114.98	153	565	12	0.600
	Delay	359.44	144.40	200	644	9	

*P*-values show the results from independent t-tests to detect differences between typically developing (TD) individuals (*N* = 22), individuals with an autism spectrum disorder (ASD) (*N* = 22), individuals with ASD who had no delay of language onset (NLD) (*N* = 12), and individuals with ASD who had a delay of language onset (LD) (*N* = 10). TD and ASD as well as NLD and LD. * Denotes *p* < 0.05.

The results of independent *t*-tests, with the Welch–Satterthwaite method used when the group variances were unequal according to the Levene’s tests, showed that the TD and ASD groups were matched on age, gender, handedness, and on the following measures: verbal, performance, and full scale IQ measured by the WASI (Wechsler, 1999); pseudoword reading, and reading comprehension measured by the WIAT-II (Wechsler, 2005), and the TROG-E (Bishop, 2005). The TD group performed significantly better than the ASD group on the BPVS-II (Dunn et al., 1997) [*t*(29.54) = 2.52, *p* = 0.017, *r* = 0.42] and the word reading subtest of the WIAT-II (Wechsler, 2005) [*t*(35.60) = 2.07, *p* = 0.046, *r* = 0.32]. LD and NLD groups were matched on all the language and cognitive ability assessment scores, as well as by age and gender. The two ASD groups differed significantly from each other in terms of handedness, as there were more left-handed individuals in the LD group [*t*(13.37) = −2.23, *p* = 0.001, *r* = 0.52]. Right-handed and left-handed TD individuals were matched on age, gender and all of the IQ and language measures. There were no participants excluded from the study after matching.

This study conforms to the Code of Ethics of the World Medical Association (Declaration of Helsinki) and was given favorable ethical opinion by the NHS Oxfordshire Research Ethics Committee C. Written consent was obtained from all the participants. Moreover, written assent was taken from all the participants who were under 16 years of age (four in the TD and six in the ASD group) accompanied by written consent from their parent/caretaker. Participants were reimbursed for their time and travel expenses.

### Experimental Design

This experiment was based upon the context-ambiguity-probe paradigm (Van Petten, 2002), which was designed to investigate the extent to which sentence context influences the processing of ambiguous sentence final words. In this approach, presented sentences end with an ambiguous word (i.e., a homonym), and the context biases either the dominant (i.e., the more common) or the subordinate (i.e., the less common) meaning of the homonym. Each sentence is followed by a related or unrelated probe word, and the stimulus onset asynchrony (SOA) between the sentence final word and the probe word is manipulated (Van Petten, 1993); the probe words follow each sentence after a short (200 ms) or long (700 ms) SOA. Probe words are related either to the subordinate or DH of the homonym and therefore are related or unrelated to the meaning of the whole sentence context. Van Petten (1993) suggested that manipulation of the SOA is a good way of estimating “the time it takes to process a prior context to a level where it may be applied to subsequent words.” Van Petten also hypothesized that when probe words are presented after a short delay, both meanings of the ambiguous word are still active and can influence the processing of the probe word. By contrast, when there is a long delay before presentation of the probe word, only the contextually relevant meaning of the ambiguous word remains active, and so the contextually irrelevant meaning does not affect processing of the probe word (Van Petten, 2002). Note that a variant of the context-ambiguity-probe paradigm was used in an earlier study by our group (Bailey et al., 2000), when only the sentences biasing the subordinate meaning (SH) of sentence final homonyms were presented.

In the current study, participants read 300 sentences presented as a sequence of words back-projected onto a translucent screen. Each sentence ended in either a homonym or an unambiguous word. There were two types of homonym sentences: in one, the homonym was used with its DH, i.e., the most common meaning; in the other, the homonym was used with its SH, i.e., the less common meaning. Each sentence was followed by a probe word. Participants were instructed to decide whether or not the probe word was semantically related to the meaning of the sentence, and to indicate their choice after a visual cue using one of two response pads.

### Final Words

#### Homonyms

An initial list of 220 homonyms was created and twelve adult (six female) native English speakers, different from the ones who participated in the main study, were asked to write down the dominant and the SHs for each homonym as they came to mind, without seeing any choice options. The 84 homonyms for which participants provided only two meanings, which were both nouns, and for which at least 7 of the 12 participants provided the same DH for the homonym were selected to be included in the study. Each homonym was used on average 2.38 (±0.48, range = 2–3) times. The average number of letters was 4.50 (±1.06, range = 3–8) in dominant homonyms and 4.55 (±1.06, range = 3–8) in subordinate homonyms, which were not significantly different (*p* > 0.05).

#### Unambiguous Words

There were 100 unambiguous final words, which were all nouns with one meaning. Each unambiguous word was used once only. The average number of letters in the unambiguous words was 5.29 (±1.25, range = 3–9), which was significantly longer than both types of homonyms (*p* < 0.05).

The word-form frequencies of all the homonyms and the unambiguous final words were obtained from the Centre for Lexical Information (CELEX) database (Baayen et al., 1995). Results of independent *t*-tests indicated that all three word types were matched (*p* > 0.05) on all frequency types (spoken, written, overall).

### Probe Words

All the probe words were nouns that had only one meaning, chosen by the authors. Each probe word was used once only. The average number of letters in a probe word was 5.35 (±1.60, range = 3–11).

### Sentences

A set of 300 sentences was prepared. Each sentence ended with either a homonym or an unambiguous word. One hundred sentences biased the DH of the homonym (e.g., The thief ran out of the bank.); one hundred sentences biased the SH of the homonym (e.g., The fisherman sat by the bank.); and one hundred sentences ended with an unambiguous word (e.g., Sally went to the cinema.). Each sentence context was semantically independent from the other sentence contexts. Every sentence was followed by a probe word that was either semantically related (e.g., The thief ran out of the bank. – money) or unrelated (e.g., The fisherman sat by the bank. – money) to the meaning of the sentence. For homonym sentences, when a probe word was semantically unrelated to the meaning of the sentence, it was related to the other meaning of the homonym that was not biased by the sentence.

In order to estimate the cloze probability of the experimental sentences, 10 native British English speakers (six female), different from the participants who helped with the creation of the homonym list and also different from the ones participated in the current main study, read the 300 sentences (without the probe word) word by word on a computer screen. Participants indicated their judgments about the expectancy of the final word on a 1–9 scale (9 for highest expectancy). Participants responded at the end of each sentence by immediately pressing a numerical key on the keyboard. Dominant homonyms had the highest expectancy followed by unambiguous words and then subordinate homonyms. A one-way ANOVA was conducted and the results indicated a significant difference between the expectancy scores for the three types of final words [*F*(2,297) = 15.18, *p* < 0.001, *r* = 0.30]. Tukey *post hoc* comparisons of the three groups indicated that the expectancy scores for dominant homonym final words (*M* = 6.35 ± 1.45) and unambiguous final words (*M* = 6.23 ± 1.50) were both significantly higher than the expectancy scores for subordinate homonym final words (*M* = 5.29 ± 1.52), *p* < 0.001. Comparisons between dominant homonym and unambiguous final words were not statistically significant at *p* < 0.05.

#### Stimulus Presentation

The experiment took place at the former Oxford Neurodevelopmental Magnetoencephalography Centre. Participants sat upright in the MEG scanner and silently read the sentences and probe words back-projected onto a translucent screen. Sentence order was randomized for each participant.

Each sentence was presented word by word, with each word displayed for 300 ms with an interval of 400 ms between the words. The mean number of words in a sentence was 5.95 (±0.86, range = 4–8). On average, there were 3.89 (±1.82, range = 1–12) letters in a word. In the current study, each sentence was followed by a probe word displayed for 800 ms that was either semantically related or unrelated to the meaning of the sentence. The probe words appeared after a short (200 ms) or a long delay (800 ms) following the final word, resulting in a SOA of 500 ms for the short and 1100 ms for the long delay, in order to avoid a large temporal overlap between the processing of final and probe words. A visual cue (two vertical bars) appeared 1600 ± 100 ms after the onset of the probe word. Participants gave their responses once they saw the cue. A red fixation square (sized 4 by 4 pixels) was presented after the cue for 2000 ± 200 ms. Stimulus words subtended a horizontal visual angle of 1.6° and a vertical visual angle of 0.4° at the eye.

The sentence words were printed in blue and the probe words in red and both were presented on a light-gray background. Each word was printed in 46-point bold Courier font. The first letters of the first words and the first letters of any proper nouns were printed in capital letters. To indicate the ending of a sentence, a period followed each final word.

Participants were instructed to indicate whether the probe word was related or unrelated to the meaning of the sentence by pressing a key with their right index finger for a related probe word, and another key with their left index finger for an unrelated probe word. Participants were instructed to respond after the visual cue, to control for motor artifacts, and asked not to blink during the presentation of final and probe words. Participants were familiarized with the scanner and testing environment in a mock scanner and room. Short or long sightedness was corrected using lenses in non-metallic frames. Before the start of the actual experiment, every participant saw a set of 12 practice sentences of the same type and was given the chance to ask questions regarding the experiment. Data from the practice sentences were not recorded but the experimenter followed the participant’s responses visually and made sure that the task was understood by each participant and that they felt comfortable using the response pad before proceeding onto the real experiment. The stimuli were presented by Stim2 software that recorded accuracy of the participant’s responses. Accuracy on the task was compared between the different participant groups and between males and females using independent *t*-tests. Reaction time data was not recorded.

#### Data Acquisition

Neuromagnetic responses were recorded using an Elekta Neuromag-306 VectorView^®^ system with a helmet-shaped array of 102 pairs of orthogonal, first-order planar gradiometers. The detectors cover the entire surface of the cortex and provide readings of orthogonal magnetic field gradients as a function of time. The output of each detector pair is most sensitive to tangential current flow in the cortical region directly below the detectors. The local root-mean-square (RMS) signal summed over the two readings is a measure of the current strength (Hamalainen et al., 1993; Braeutigam et al., 2004). Averaged evoked fields for final and probe words were recorded. The data were sampled at 1000 Hz (0.03–330 Hz anti-alias filter). Vertical EOG (electrooculogram) and ECG (electrocardiogram) signals were recorded during the experiment to control for artifacts. Each participant’s head coordinates were measured by registering the right and left preauricular points, as well as the nasion. Head digitization was established by recording the position of four Head Position Indicator (HPI) coils and additional digitization points were taken over the whole head in order to improve the accuracy of data pre-processing. An HPI measurement was taken at the start of each testing session and all participants were positioned in approximately the same position in the helmet. Head movements were monitored visually via a camera system and if there was a movement the HPI measurement was repeated to ensure that the participant’s head was back in the same location.

After every 25 sentences, participants had a short break (approximately 10 s). The experiment lasted approximately 50 min and participants completed the task in two halves, coming out of the scanner for a 10-min break after 150 sentences. At the beginning of the second half of the experiment, the HPI measurement was repeated and head position adjusted to be within the ±5 mm of the initial location.

#### Data Pre-processing

Each data set was spatially filtered using Neuromag^®^ MaxFilter 2.0 software to reduce interference arising outside the head. MaxFilter^TM^ with temporal extension was used to reduce far-field interference and artifacts due to dental works. Independent EOG and ECG recordings in conjunction with standard projection algorithms were used to correct for artifacts related to eye-blinks and heart activity. The MEG signals of each participant were transferred to a reference set of head coordinates, chosen separately for TD and ASD groups because of possible different average head sizes in each participant group, using MaxFilter to ensure standardization within each participant group. The total number of trials for each condition across all participant groups were the same.

#### Time-Series Analysis

Data from the two experimental halves were averaged into one dataset for each participant and the averaged evoked fields for final words were analyzed. The delay between the computer triggers and appearance of the stimuli on the screen was measured as 10 ms, which was corrected for in the analyses.

Evoked responses were averaged separately for each of the three final word types at the short and long SOAs. In this paradigm, probe words at long SOA were presented 1100 ms after the onset of final words and there was no overlap between the neural responses to the final words and probe words. Probe words at short SOA were presented only 500 ms after final word onset, creating some overlap between the neural responses to final words and probe words. Thus, we report results only until 500 ms after final word onset. Average signals were filtered using a 30 Hz low-pass filter. Each evoked response was baseline corrected (mean value) using data from 100 ms before stimulus onset.

In this paper, we report the responses to final words in order to focus on the effects of sentence context on the semantic processing of different types of final words varying in their level of ambiguity. We present results from two main time windows: 130 – 200 ms and 350 – 500 ms (N400 interval) for sentence final words (see also Supplementary Figures 1A,B).

For an initial visual inspection of the data, global (all gradiometer channels) RMS curves were calculated for each word condition for each participant and for both participant groups. To identify the spatial distribution of activity for each word condition, local RMS-maps were prepared for each participant and for both participant groups. The local RMS signal represents the measure of the current strength in the region directly below the detectors (Hamalainen et al., 1993; Braeutigam et al., 2004, 2008). Local maps of activity are presented for different word conditions for the latencies where the activation was strongest within the identified time window.

Differences in the evoked responses to different word categories were sought using a time-dependent, chi-square-based measure P(*t*) (Hamalainen et al., 1993; Braeutigam et al., 2004, 2008), which takes into account the data from all sensors and does not require *a priori* identification of peaks in the evoked responses. The approach aims to obtain a robust measure (P) of the significance of the differences between evoked responses in signal space as a function of latency.

$$P\,\left(t\right)=\mathrm{probability}\,\left(\chi^{2}\right),\text{with}\,\chi^{2}=-2\,\sum_{i=1}^{N}\text{In}\,\left[f_{i}\,\left(t\right)\right]$$

In this formula, *N* denotes the number of channels (here: 204) and *f*_*i*_(t) denotes a non-parametric statistical test. Note that a time point is significant only if the cumulative chi-square test is significant, implying a correction for multiple comparisons across channels. For the purpose of this paper, time points with P(*t*) < 0.01 were considered as significant. For each significant interval, the set of all values *f*_*i*_ provided the spatial distribution of the significance of the differences between evoked responses. These distributions were used to identify significant regions at the group level. For each significant latency, maps of distribution of significance were prepared. Note that not all significant areas relate to easily identifiable changes in signal power (neural activity). This is because MEG [as well as electroencephalography (EEG)] can detect changes in neuronal dynamics that are not, or only to a small degree, associated with power changes.

Three different statistical tests (*f*_*i*_) were used to analyze the within and between group differences in response to different words conditions. A Friedman analysis of variance was used to determine if there was a significant difference among the three final word conditions within a certain time window in each participant group. When the Friedman analyses were significant, *post hoc* tests of Wilcoxon identified between which of the two final word conditions there was a significant difference. Lastly, we conducted Mann–Whitney *U* tests to establish if there were significant differences between two participant groups for each of the final word conditions. After the initial analyses of the differences between the neural responses in the TD and ASD groups, we divided the ASD group into two subgroups (NLD and LD) to examine the correlates of a history of language delay in ASD with word understanding during sentence reading. These tests and their results, which are represented on the maps of distribution of significance, are bi-directional. Once significance was detected, the local maps of activity were inspected to determine the directionality of power differences. The analyses were conducted in sensor space as insufficient anatomical MRI scans were available.

An ancillary Mann–Whitney *U* analysis between the right-handed LD (*N* = 5) and left-handed LD (*N* = 5) participant groups was carried out in order to exclude a possible confound due to unequal numbers of left- and right-handers in the LD and NLD groups (five of the six left-handed participants with ASD belonged to the LD group).

## Results

All participants completed the task successfully with an average accuracy of 79.11%. The TD group had higher accuracy scores (83.75%) than the ASD group (77.39%), but this difference did not reach significance, and there were no significant differences in performance between the NLD (74.63%) and LD (80.71%) ASD sub-groups. There were also no significant differences between the male and female participants in any of the participant groups.

**FIGURE 1:**
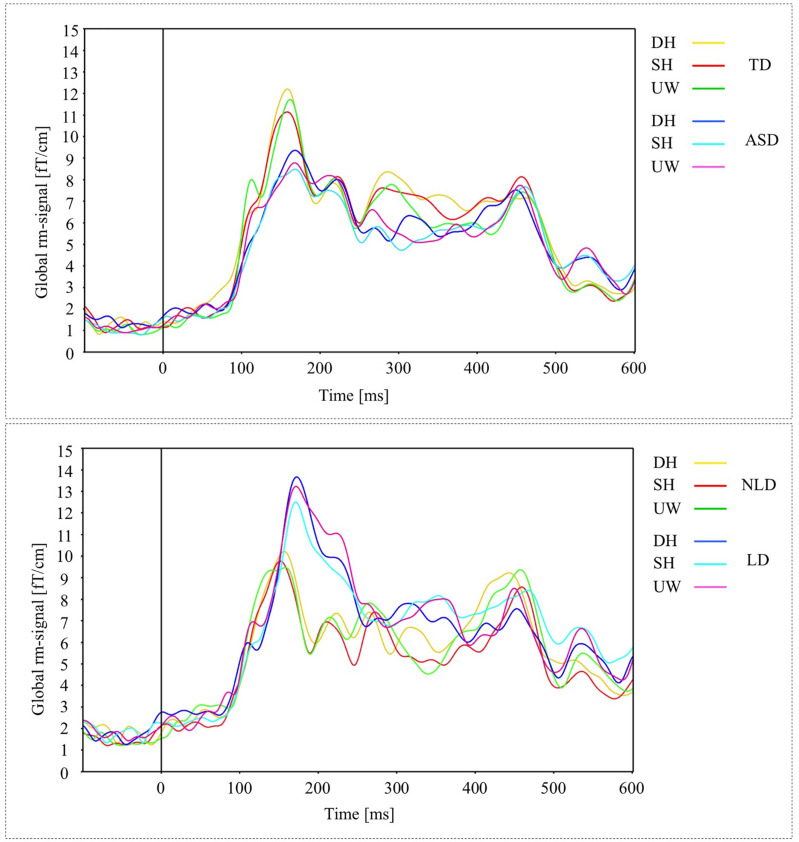
**(A)** Global activations (RMS-curves) for three final words (dominant homonym, subordinate homonym, and unambiguous word) in TD and ASD groups. **(B)** Global activations (RMS-curves) for three final word conditions in NLD and LD groups.

A preliminary analysis of the MEG data revealed a time course of brain activation (as measured by the global RMS-signals) that was broadly similar across all participants and final word conditions (Figures 1A,B). Evoked activity was first observed at about 100 ms, predominantly over primary visual cortices. This response was followed by strong activity at about 150 ms over mainly midline parietal and left inferior occipito-temporal cortices. Subsequent activity up to about 300 ms was widespread and without any obvious pattern that could be identified visually. Response around 350 ms was stronger for all final words in the TD group compared to the ASD group. At longer latency, N400-like peaks in neuronal activity were consistently observed in all conditions, predominantly over left parieto-temporal and right posterior parietal cortices. The strongest activity was observed at 450 ms, whereas the significant differences between stimuli were observed around 400 ms. Typically, the effects of word condition on evoked power were small (≤5% power difference) at all latencies within each participant group.

In general, the measure P(*t*) revealed significant intervals to varying degrees throughout the whole post-stimulus interval, dependent on participant group and stimulus condition. Given the present data, however, it was not possible to interpret reliably all significant effects, and further analysis was restricted to latencies around 150 and 400 ms, corresponding to maximal evoked power and the N400 window respectively. In keeping with the study objectives, and in order to reduce the large parameter space provided by experiments of this kind, the analysis focused on within-group, matched sample comparisons. In the remainder of this section, a detailed description is given of significant effects on the neuronal responses to final words. In order to complement the within-group approach, a number of ancillary between group analyses were carried out using Mann–Whitney *U* tests.

### Sentence Final Words

#### 130 – 200 ms

##### TD Group

**FIGURE 2:**
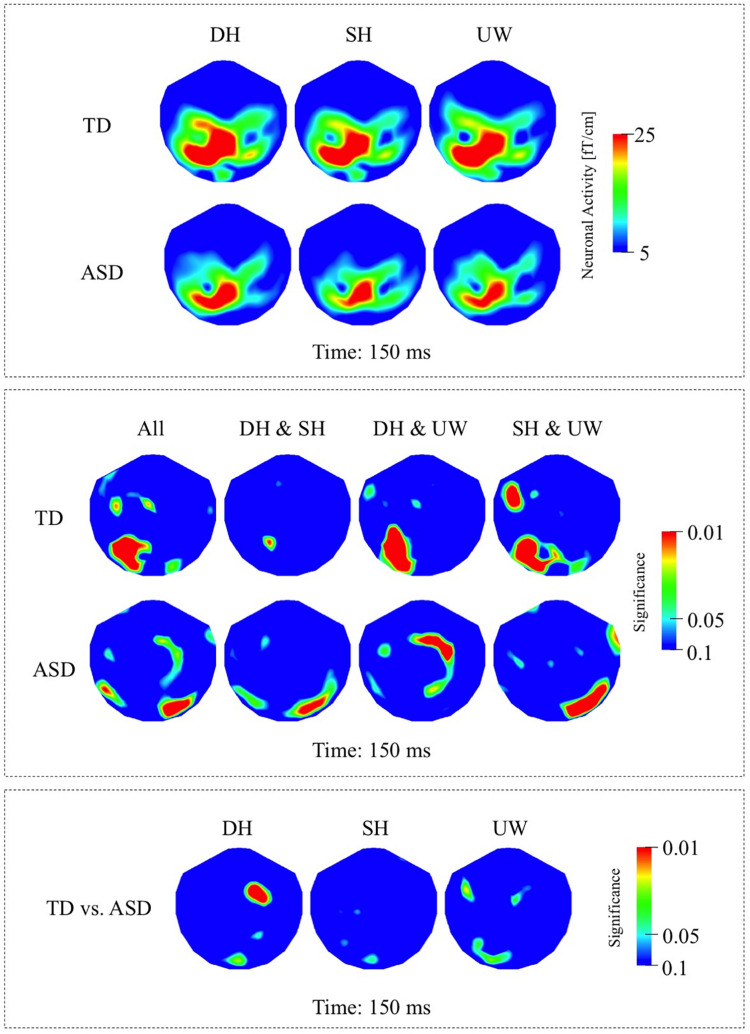
**(A)** First row: Local RMS-maps for the TD group’s responses to final words (dominant homonym, subordinate homonym, and unambiguous word) at 150 ms. Second row: Local RMS-maps for the ASD group’s responses to final words (dominant homonym, subordinate homonym, and unambiguous word) at 150 ms. **(B)** First row: TD group’s Friedman analysis result for dominant homonym, subordinate homonym, and unambiguous word at 150 ms. Wilcoxon pairwise analysis result of dominant homonym and subordinate homonym, dominant homonym and unambiguous word, and subordinate homonym and unambiguous word at 150 ms. Second row: ASD group’s Friedman analysis result for dominant homonym, subordinate homonym, and unambiguous word at 150 ms. Wilcoxon pairwise analysis result of dominant homonym and subordinate homonym, dominant homonym and unambiguous word, and subordinate homonym and unambiguous word at 150 ms. **(C)** Mann–Whitney *U* analysis result for TD vs. ASD groups’ responses to final words (dominant homonym, subordinate homonym, and unambiguous word) at 150 ms. For the presentation of data, the detectors have been projected into two dimensions (right ear on the right, front at the top).

The responses evoked by the three types of final word were observed over the left occipito-parietal and left posterior temporal regions (Figure 2A, first row). The responses evoked by the unambiguous word condition were significantly greater than for both the dominant and subordinate homonym conditions over the left occipito-temporal regions (Figure 2B, first row).

##### ASD Group

The final word evoked responses occurred mainly over the left occipito-parietal regions, with reduced activity over left posterior temporal regions compared to the TD group (Figure 2A, second row). The response to the subordinate homonym condition was significantly stronger than the responses to the dominant homonym and unambiguous word conditions over the right occipito-temporal regions. Dominant homonyms evoked significantly stronger responses than the unambiguous words over the right parietal regions (Figure 2B, second row).

##### TD vs. ASD Groups

The Mann–Whitney *U* analyses showed significantly stronger activity in the TD group than the ASD group for the dominant homonyms over the right fronto-parietal regions (Figure 2C).

##### NLD Group

**FIGURE 3:**
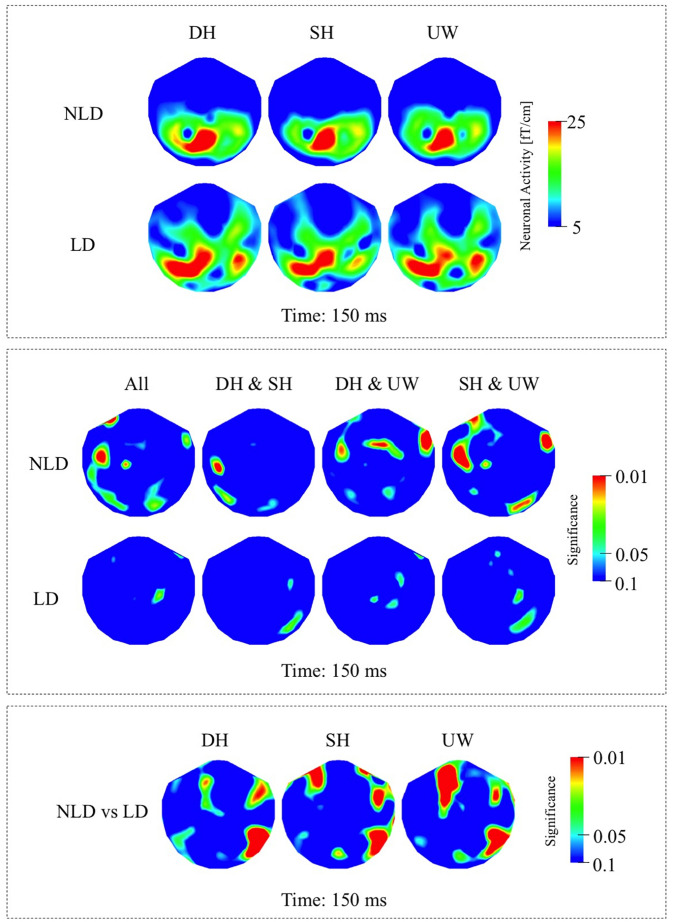
**(A)** First row: Local RMS-maps for the NLD group’s responses to final words (dominant homonym, subordinate homonym, and unambiguous word) at 150 ms. Second row: Local RMS-maps for the LD group’s responses to final words (dominant homonym, subordinate homonym, and unambiguous word) at 150 ms. **(B)** First row: NLD group’s Friedman analysis result for dominant homonym, subordinate homonym, and unambiguous word at 150 ms. Wilcoxon pairwise analysis result of dominant homonym and subordinate homonym, dominant homonym and unambiguous word, and subordinate homonym and unambiguous word at 150 ms. Second row: LD group’s Friedman analysis result for dominant homonym, subordinate homonym, and unambiguous word at 150 ms. Wilcoxon pairwise analysis result of dominant homonym and subordinate homonym, dominant homonym and unambiguous word, and subordinate homonym and unambiguous word at 150 ms. **(C)** Mann–Whitney *U* analysis result for NLD vs. LD groups’ responses to final words (dominant homonym, subordinate homonym, and unambiguous word) at 150 ms. For the presentation of data, the detectors have been projected into two dimensions (right ear on the right, front at the top).

In the NLD group, evoked responses were most strongly observed over the left occipito-parietal and left posterior temporal regions in all final word conditions (Figure 3A, first row). Responses evoked by dominant homonyms were significantly greater than the responses evoked by the unambiguous words over the right anterior temporal regions. Subordinate homonyms evoked significantly greater responses than unambiguous words over the right occipito-temporal regions (Figure 3B, first row).

##### LD Group

In the LD group the responses evoked by all final word types were observed strongly over the left posterior temporo-parietal regions and to a varying degree over the right posterior temporal regions (Figure 3A, second row). According to measure P(*t*), the evoked responses at this latency were not significantly modulated by the final word condition (Figure 3B, second row).

##### NLD vs. LD Groups

As indicated by the Mann–Whitney *U* analyses, in all three final word conditions, the evoked responses over the right occipito-temporal regions were significantly stronger in the LD compared to the NLD group. Also, evoked activity in the LD group was, to varying degrees, significantly stronger over the right anterior temporal regions and over the left frontal regions than in the NLD group in all three final word conditions (Figure 3C).

#### 350 – 500 ms (N400 Interval)

##### TD Group

**FIGURE 4:**
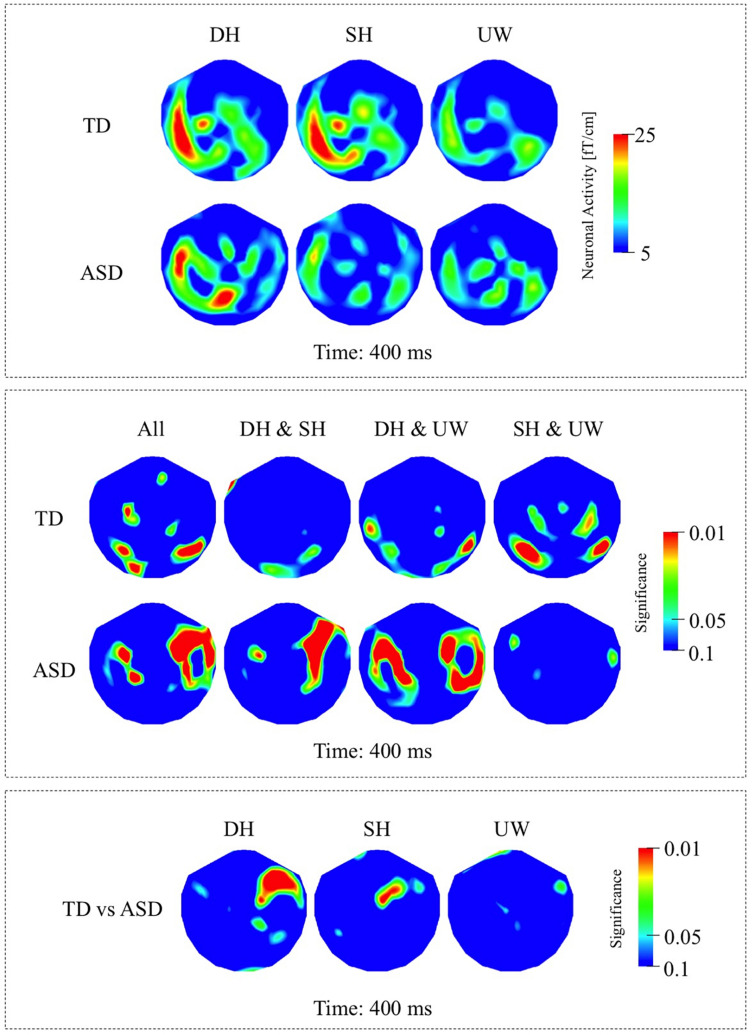
**(A)** First row: Local RMS-maps for the TD group’s responses to final words (dominant homonym, subordinate homonym, and unambiguous word) at 400 ms. Second row: Local RMS-maps for the ASD group’s responses to final words (dominant homonym, subordinate homonym, and unambiguous word) at 400 ms. **(B)** First row: TD group’s Friedman analysis result for dominant homonym, subordinate homonym, and unambiguous word at 400 ms. Wilcoxon pairwise analysis result of dominant homonym and subordinate homonym, dominant homonym and unambiguous word, and subordinate homonym and unambiguous word at 400 ms. Second row: ASD group’s Friedman analysis result for dominant homonym, subordinate homonym, and unambiguous word at 400 ms. Wilcoxon pairwise analysis result of dominant homonym and subordinate homonym, dominant homonym and unambiguous word, and subordinate homonym and unambiguous word at 400 ms. **(C)** Mann–Whitney *U* analysis result for TD vs. ASD groups’ responses to final words (dominant homonym, subordinate homonym, and unambiguous word) at 400 ms. For the presentation of data, the detectors have been projected into two dimensions (right ear on the right, front at the top).

Within the N400 time window, the strongest responses were observed at 450 ms; however, significant differences among the different final words were observed slightly earlier at 400 ms. All three final word conditions evoked strong responses over the left occipito-parietal, left temporal, and to varying degrees over the right posterior temporal regions (Figure 4A, first row; Figure 6A, first row). Dominant homonyms responses were significantly stronger than unambiguous word responses over the left occipital regions. Subordinate homonyms revealed significantly stronger responses over the left occipital regions than the dominant homonyms and over the left temporal regions than the unambiguous words. Responses to unambiguous words were significantly stronger than the responses to dominant and subordinate homonyms over the right posterior temporal regions (Figure 4B, first row).

##### ASD Group

While the strongest response was observed at 450 ms, the significant differences among the three final word conditions within the N400 time window were observed at 400 ms. Activity was mainly localized over the left occipito-parietal and, to varying degrees, over the bilateral temporal regions for all three final words (Figure 4A, second row; Figure 6A, second row). Responses evoked by dominant homonyms were significantly greater than the responses evoked by subordinate homonyms and unambiguous words over the right parieto-temporal regions. Moreover, dominant homonyms had significantly greater responses than both the subordinate homonyms and unambiguous words over the left temporal regions (Figure 4B, second row).

##### TD vs. ASD Groups

Mann–Whitney *U* analyses showed stronger responses over the right fronto-temporal regions in the ASD group than the TD group in dominant homonym and subordinate homonym final word conditions (Figure 4C).

##### NLD Group

**FIGURE 5:**
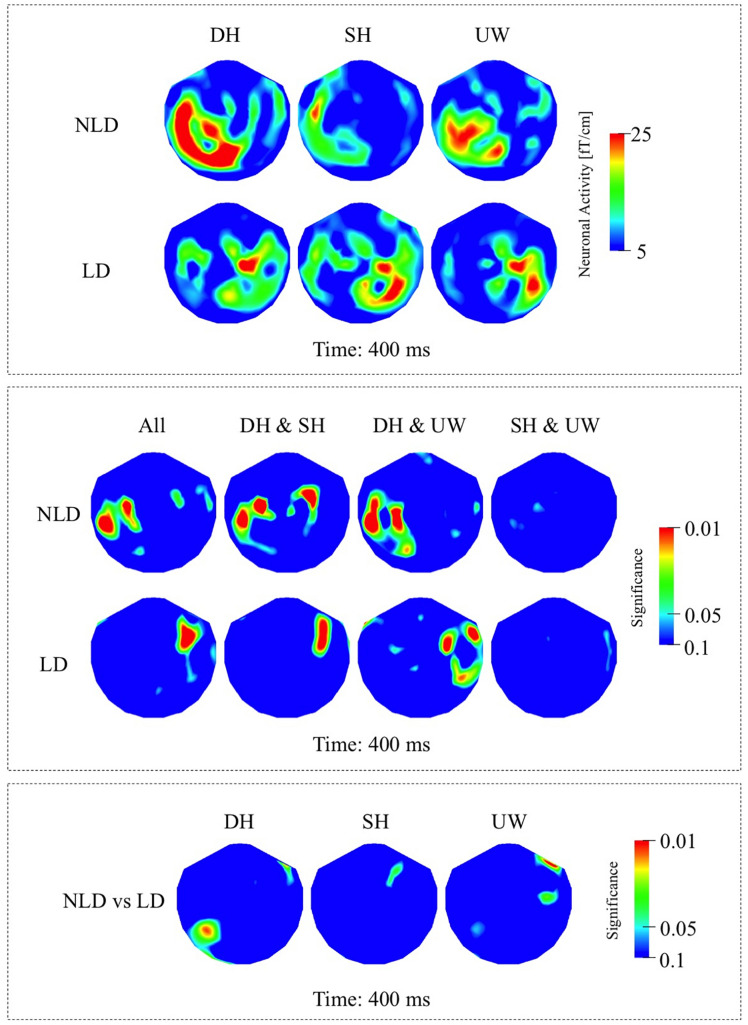
**(A)** First row: Local RMS-maps for the NLD group’s responses to final words (dominant homonym, subordinate homonym, and unambiguous word) at 400 ms. Second row: Local RMS-maps for the LD group’s responses to final words (dominant homonym, subordinate homonym, and unambiguous word) at 400 ms. **(B)** First row: NLD group’s Friedman analysis result for dominant homonym, subordinate homonym, and unambiguous word at 400 ms. Wilcoxon pairwise analysis result of dominant homonym and subordinate homonym, dominant homonym and unambiguous word, and subordinate homonym and unambiguous word at 400 ms. Second row: LD group’s Friedman analysis result for dominant homonym, subordinate homonym, and unambiguous word at 400 ms. Wilcoxon pairwise analysis result of dominant homonym and subordinate homonym, dominant homonym and unambiguous word, and subordinate homonym and unambiguous word at 400 ms. **(C)** Mann–Whitney *U* analysis result for NLD vs. LD groups’ responses to final words (dominant homonym, subordinate homonym, and unambiguous word) at 400 ms. For the presentation of data, the detectors have been projected into two dimensions (right ear on the right, front at the top).

**FIGURE 6:**
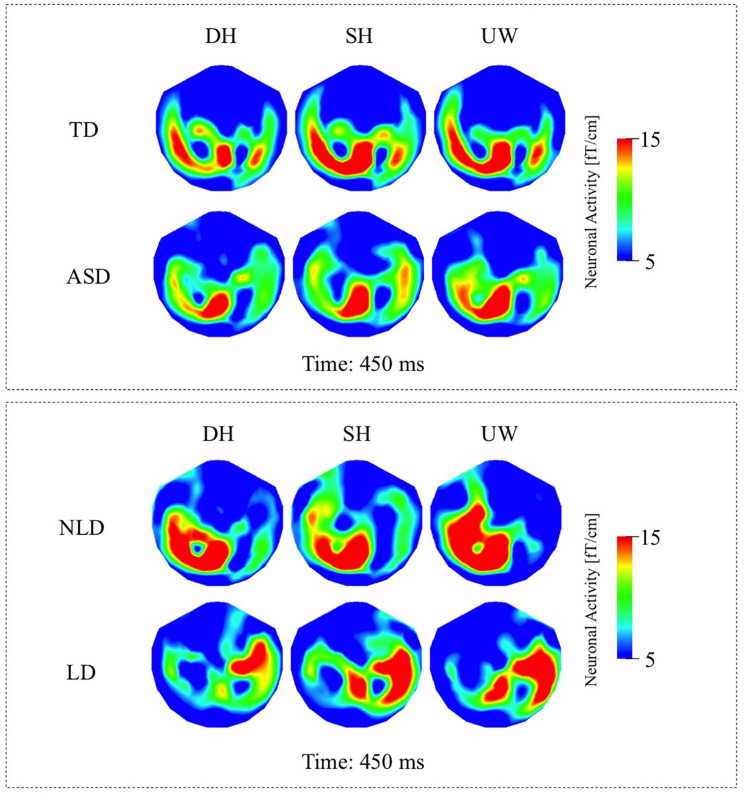
**(A)** Top row: TD group’s local RMS-maps for dominant homonym, subordinate homonym, and unambiguous word at 450 ms. Bottom row: ASD group’s local RMS-maps for dominant homonym, subordinate homonym, and unambiguous word at 450 ms. **(B)** Top row: NLD group’s local RMS-maps for dominant homonym, subordinate homonym, and unambiguous word at 450 ms. Bottom row: LD group’s local RMS-maps for dominant homonym, subordinate homonym, and unambiguous word at 450 ms. For the presentation of data, the detectors have been projected into two dimensions (right ear on the right, front at the top).

While the strongest N400 responses were observed at 450 ms, the significant differences among the final words were observed at 400 ms. In the NLD group, evoked responses were observed over the left occipito-parietal and left posterior temporal regions for all three final words (Figure 5A, first row; Figure 6B, first row). The responses to the dominant homonyms were stronger compared to the subordinate homonyms and unambiguous words over the left parieto-temporal regions (Figure 5B, first row).

##### LD Group

The strongest responses within the N400 time window were observed at 450 ms, while the significant differences among the final words were observed at 400 ms. In the LD group, evoked responses were localized over the right fronto-temporal regions and to varying degrees over the left temporal regions for all three final words (Figure 5A, second row; Figure 6B, second row). The evoked responses within the N400 time window were not significantly modulated by the final word condition (Figure 5B, second row).

##### NLD vs. LD Groups

Mann–Whitney *U* analyses revealed that the evoked responses to dominant homonyms were significantly stronger in the NLD group than the LD group over the left posterior temporal regions (Figure 5C).

### Comment on Handedness

At 150 ms, Mann–Whitney *U* analysis results revealed a significant difference between the left-handed LD and right-handed LD groups for the SH and UW final word conditions where the increased right-sided activity was stronger in the left-handed LD group, but there were no significant differences between the two LD groups for the DH final word condition. At the N400 latencies there were no significant differences for any of the final word conditions between the two LD groups (Supplementary Figures 2A–C).

## Discussion

This study examined the neuronal correlates of semantic ambiguity resolution during reading in individuals with ASD and in TD individuals whose current language functioning was extremely well characterized. As an extension of previous work (Bailey et al., 2000; Ahtam, 2009), this study describes the responses to sentence final dominant and subordinate homonyms as well as unambiguous words and investigates the association between early language delay and semantic processing anomalies in individuals with ASD. The main findings of this study are evidence for semantic access at 150 ms and N400 latencies in TD and ASD individuals with no history of language delay. We found reduced sensitivity to contextual information at 150 ms (Figure 3B, second row) and also strong right hemisphere lateralization at both latencies (Figure 3A, second row; Figure 5A, second row) in individuals with ASD who had a history of LD. Crucially; the current tested language abilities of the LD and NLD groups with ASD were indistinguishable. These results provide new evidence and support for differential neural mechanisms underlying semantic processing in ASD, and show that delayed language acquisition in ASD is associated with different processing and hemispheric lateralization of language that is not necessarily reflected in performance on language tests.

### Response at 130 – 200 ms

In this study, the final word data at 150 ms in both the TD and NLD groups are consistent with suggestions that semantic information is accessed as early as 150–180 ms after visual presentation of a word (Penolazzi et al., 2007; Shtyrov and Pulvermuller, 2007). At this latency both groups processed the difference between a homonym and an unambiguous word, although this processing may involve different neuronal pathways in TD and ASD, as significant effects are absent over left posterior cortices in NLD compared to TD (Figure 2B, first row; Figure 3B, first row). Moreover, both the TD and NLD groups failed to process the difference between the two homonym types (Figure 2B, first row; Figure 3B, first row), leaving it unresolved if sentence context plays a role at this relatively early latency. By contrast, the lack of any difference between the evoked responses to the three types of final word in the LD group (Figure 3B, second row) suggests that they had not processed the difference between ambiguous and unambiguous words at this early latency. Conceivably, these findings are indicative of early differential processing within the ASD cohort, where neuronal processing in LD individuals is more word (as opposed to context) oriented and associated with stronger activity over right occipito-temporal cortices.

### Response at 350 – 600 ms (N400 Interval)

Previous studies investigating semantic integration found amplitude and latency differences between the N400 responses of ASD and TD groups. These findings were interpreted as indicating less elicitation of alternative meanings of ambiguous words (Strandburg et al., 1993), changes in expectancy for the upcoming word stimuli in context (Dunn et al., 1999; Dunn and Bates, 2005), difficulties in integrating words rapidly to establish meaningful context (Bailey et al., 2000; Ring et al., 2007; Gold et al., 2010; Pijnacker et al., 2010), and the use of different neuronal networks in semantic processing and semantic ambiguity resolution (Valdizan et al., 2003; Braeutigam et al., 2008; McCleery et al., 2010; Ribeiro et al., 2013). In this study, the TD (Figure 4B, first row) and NLD (Figure 5B, first row) groups, but not the LD group (Figure 5B, second row), showed significant differences between the responses to dominant and subordinate homonyms in the N400 interval, suggesting that by this latency both groups used context to help distinguish the different meanings of homonyms. The precise functional significance of the responses to final words at around 400 ms is unresolved. For all conditions, the final word always matched the sentence making an interpretation in terms of semantic violation unlikely. Conceivably, the activity at N400-like latencies relates to a form of semantic closure (Bailey et al., 2000) whereby the sentence is finalized and semantic anticipation for upcoming probe words initiated. In this view, the neuronal processing indexes integration of individual word meanings into a sentence context and expectancy of a final word, where subordinate homonym words, which are by definition less frequent in everyday use, might be less expected, leading to stronger N400 responses compared to unambiguous words in particular (Kutas and Hillyard, 1984a; West and Holcomb, 2002; Lau et al., 2008).

### Hemispheric Asymmetry in ASD

Previous research suggests a strong relationship between atypical laterality of language and language impairment in ASD (Lindell and Hudry, 2013; Markou et al., 2017). Atypical hemispheric asymmetry, increased right hemispheric activity and/or decreased left hemispheric activity, in ASD is seen across different aspects of language processing, including speech processing (Dawson et al., 1986), sentence listening (Muller et al., 1999), passive listening to speech-like sounds (Boddaert et al., 2003), silent sentence reading (Takeuchi et al., 2004), passive listening to speech sounds (Flagg et al., 2005; Yoshimura et al., 2013), lexico-semantic processing (Harris et al., 2006), understanding of irony (Wang et al., 2006), semantic category decision-making (Gaffrey et al., 2007), processing of congruous and incongruous final words (Braeutigam et al., 2001, 2008), word generation (Knaus et al., 2010), and story listening during sleep (Eyler et al., 2012). Such atypical hemispheric lateralization has been observed also in non-language tasks such as passive listening to simple tone stimuli (Gage et al., 2003; Edgar et al., 2015; Berman et al., 2016). Abnormal rightward lateralization has been found both in very young (1–4 years old) children with autism (Eyler et al., 2012), as well as in 3–7 year old children with autism (Yoshimura et al., 2013), reflecting an early developmental pathology (Redcay and Courchesne, 2008; Eyler et al., 2012), although the relationship with language development was not reported.

In this study, significant effects were more right lateralised in individuals with ASD compared to TD participants (Figures 2B, 4B). Consistent with the distribution of significance, neuronal responses were less (left) lateralised in individuals with ASD compared to TD, where particularly strong responses were observed over the right hemisphere in ASD individuals, which is a finding in parallel with previous neuroimaging and neurophysiological studies of autism (Dawson et al., 1986, 1989; Takeuchi et al., 2004; Flagg et al., 2005; Braeutigam et al., 2008; Seery et al., 2013). Despite the lack of difference in the responses to final words in the LD participants (Figure 3B, second row), significant differences between the two ASD groups during the earlier 130–200 ms time window (Figure 3C) suggest that right hemispheric activity was increased in the LD compared to the NLD group, in particular over right occipito-temporal cortices.

It is commonly assumed that the cerebral hemispheres have different roles in integrating word meanings into context. The left hemisphere is biased toward prediction, where on-going context processing leads to expectancy for upcoming words, and new information is compared with expectation rather than actual context (Federmeier and Kutas, 1999). By contrast, the right hemisphere is biased toward evaluation of new information and its integration with context (Federmeier and Kutas, 1999). The increased right-sided activity observed in the LD group suggests that these individuals may activate atypical neural networks during early stages of ambiguity resolution that is abnormally centered on individual words rather than sentence context (Harris et al., 2006; Braeutigam et al., 2008). This finding is supported by behavioral and EEG studies that found that individuals with autism had greater difficulty in using contextual information than individuals with Asperger’s syndrome (Jolliffe and Baron-Cohen, 1999, 2000; Pijnacker et al., 2010). Consequently, individuals with ASD might find it challenging to establish a normal balance between expectation and integration, which may entail a more piecemeal, word-oriented approach to language processing. Ultimately, it seems, atypical semantic strategies are present in individuals with ASD who participated in our study and performed as well as the TD individuals. In some individuals with ASD, such strategies might be generally sufficient for language comprehension and production but might create difficulties when ambiguity resolution at different levels (word, sentence, discourse) is required, leading to specific anomalies in the use of language.

### History of Delayed Language Onset in ASD

To our knowledge, this is the first MEG study to show increased right hemispheric response in a semantic task in individuals with ASD who had delayed language onset versus individuals with ASD without delayed language. It is possible that the increased right hemispheric lateralization seen in previous studies was also related to a history of language delay in individuals with ASD. It is unclear, however, whether the development of different semantic networks caused, or was caused by, a delay in language onset. Another possibility is that the development of aberrant semantic networks and a delay in language onset could both have been caused by a third factor, genetic predisposition. All studies show that there is considerable genetic heterogeneity in ASD (Chaste and Leboyer, 2012), therefore it is very likely that multiple physiological processes are affected.

### Weak Central Coherence Theory

The assumption of weak central coherence theory, which argues that individuals with ASD have difficulties integrating individual word meanings into a meaningful sentence context (Frith and Snowling, 1983; Frith, 1989) was not met by this experiment. The relatively high accuracy scores in both participant groups implies that both the TD and ASD participants were able to integrate individual word meanings into a meaningful sentence context to decide on the relatedness of the probe words. The findings from this study suggest that semantic processing difficulties in individuals with ASD are instead related to either weaker activation of the SH or stronger activation of the DH of ambiguous words and delayed influence of sentence context on semantic access of individual words. These findings have implications for the understanding of language in a broader context, such as ambiguity in daily speech and the difficulties individuals with ASD experience with interpreting allusive language, metaphors, idioms, and jokes (Happé, 1994). Our results are consistent with the findings of previous studies that used other measures such as semantic priming, eye-tracking, and reading time (Lopez and Leekam, 2003; Norbury, 2005; Saldaña and Frith, 2007; Brock et al., 2008, 2017; Henderson et al., 2011), which have all shown that individuals with autism used sentence context effectively for accurate comprehension. Our results are also more in line with an alternative theory to the weak central coherence theory, which suggests that individuals with autism fail to use sentence context to select the correct phonological form of the homograph for accurate pronunciation, reflecting an impairment in word production rather than comprehension (Brock et al., 2017).

### Limitations

One of the limitations of this study is the sample size of the two ASD sub-groups. Although sample sizes for the TD and ASD groups were relatively large, once the ASD group was divided into two, the individual sample sizes for the NLD and LD groups were modest. Moreover, it would have been desirable to have similarly sized TD, NLD and LD groups to be able to conduct analyses to compare the brain activity of NLD and LD groups versus the TD group. With these analyses missing, it is unknown how the ASD subgroups differ from the TD controls. Another consideration is the relatively high number of left-handed individuals in the LD ASD sub-group. When responses of the ASD sub-groups were examined, it was clear that right-sided activity was stronger in the LD than the NLD group at both latencies (Figures 3A, 5A, 6B). Five of the six left-handed participants with ASD belonged to the LD group. In contrast, there was only one left-handed participant in the NLD group. When the LD group was divided into right- and left-handed participants, we did not observe a consistent relationship between left-handedness and increased right hemispheric activity. Note that this finding is in line with previous studies reporting increased right hemispheric activity in right-handed ASD participants (Takeuchi et al., 2004; Flagg et al., 2005; Braeutigam et al., 2008; Pijnacker et al., 2010; Markou et al., 2017). Nevertheless, it would have been desirable to have had equal numbers of right- and left-handed participants in NLD and LD, as well as TD groups. It is worth noting that in comparison with many studies in the field, the current language functioning of participants was comprehensively examined.

Even though TD and ASD groups were age matched (mean age in both groups was 20 years), another limitation could be the age range (15–33 years in TD, 14–43 years in ASD) of the participants in the current study. Nevertheless even though the oldest participant with ASD was 43 years old, the second oldest participant in the ASD group was only 33 years old. Moreover, we did not observe any clear differences between the data of the 43-year-old participant compared to the other ASD participants, neither are we aware of studies finding significant changes in the brain basis of language processing across the age range we have studied. Previous studies finding age related changes (Federmeier et al., 2002, 2003) had much wider age ranges (e.g., overall age range = 18–79 years) than in this study.

Another possible limitation concerns participants’ knowledge of both meanings of the homonyms. Because of their age and high level of abilities, all individuals who took part in this study were expected to be familiar with both meanings of the homonyms, although this was not rigorously tested. The high accuracy scores, however, indicates that participants were usually familiar with both meanings. Furthermore, despite our best efforts to match the TD and ASD participant groups on all the pre-assessments, they were not matched on their accuracy scores for the BPVS or the word reading subtest of WIAT-II, where the TD group performed better than the ASD group. On the other hand, the NLD and LD groups were matched on all measures, including the BPVS and the word reading subtest of WIAT-II, and we still observed significant differences in brain activity between the two ASD subgroups. Therefore, it seems unlikely that the overall differences observed between the TD and ASD groups could be attributable to the significant difference in their BPVS accuracy scores. It is widely acknowledged that age of language acquisition is an important variable in linguistic tasks. The age of onset for single words and simple phrases was acquired from a diagnostic interview (i.e., ADI-R) conducted with the parents of participants with ASD. No information regarding age of language onset was acquired for the TD group, however, although it was assumed to be within the normal range because of their generally typical development. Furthermore, since we only focus on the electrophysiological activity of final word responses in this manuscript, and not the probe words, we were not interested in correlating behavioral data (i.e., accuracy results for assessing the probe word relatedness to the sentence context) with electrophysiological responses to the final words which are seen and processed before the probe words are seen. Future work that focuses on the probe word responses could benefit from such correlational analyses which could give additional insight into the interpretation of the results.

In the current study the mean number of letters in the unambiguous words was significantly higher than the mean number of letters in the two homonym final word conditions but just by 0.79 letters (mean number of letters in unambiguous words = 5.29, homonyms = 4.50). Previous literature suggests stronger brain responses for longer words than shorter words (mean number of letters in long words = 6.1, short words = 3.9) (Assadollahi and Pulvermuller, 2003). Although the possibility should not be discarded, it is unlikely that the observed differences in this study were due to word length effects, because the difference in our study was on average less than one letter, whereas in the previous studies the difference was more.

Finally, due to an insufficient number of anatomical MRI scans being available, the analyses were conducted in sensor space. While the lack of data analysis at the source level limits the scope of interpretation, it does not invalidate the results that have been found in this study such as observation of absence of neuronal modulation in patients with delayed language onset. We decided against the use of a (typically developing) template brain and conducted all analyses in sensor space, because of converging evidence of (minor) physical abnormalities in ASD, such as the cephalic index (Tripi et al., 2008), which can produce misleading results when using an ‘average’ brain.

## Conclusion

In summary, the results of this study provide important insights into the use of sentence context for the activation of individual words in individuals with ASD and in TD individuals. We show differences between the LD and NLD individuals within the ASD group, where delayed language acquisition was associated with abnormally increased right hemispheric activity. Investigation of abnormal laterality might contribute to early identification of language delay in individuals at risk for an ASD leading to earlier and more efficient speech and language therapies (Lindell and Hudry, 2013). We conclude that history of language acquisition is a potential marker of physiological heterogeneity within ASD, and future studies should consider the history of language acquisition in individuals with a diagnosis of ASD, since combining data from different ASD sub-groups may hide differences between the two groups (Pijnacker et al., 2010).

## Data Availability Statement

The data for this article are not publicly available at this time. Requests to access the data should be directed to the corresponding author.

## Ethics Statement

The studies involving human participants were reviewed and approved by NHS Oxfordshire Research Ethics Committee C. Written informed consent to participate in this study was provided by the participants’ legal guardian/next of kin.

## Author Contributions

BA and SB contributed to the experimental design, data collection, data analysis, interpretation of results, and writing of the manuscript. AB contributed to the experimental design, interpretation of the results, and writing of the manuscript. All authors contributed to the article and approved the submitted version.

## Conflict of Interest

The authors declare that the research was conducted in the absence of any commercial or financial relationships that could be construed as a potential conflict of interest.

## Abbreviations

ASD: autism spectrum disorder
EEG: electroencephalography
ERP: event-related potential
LD: language delay
MEG: magnetoencephalography
NLD: no language delay
RMS: root mean square
SOA: stimulus onset asynchrony
TD: typically developing.
