# Supplementary material for: Semantic Processing in Autism Spectrum Disorders Is Associated With the Timing of Language Acquisition: A Magnetoencephalographic Study
Source: Front Hum Neurosci. 2020 Jul 10;14:267. doi: 10.3389/fnhum.2020.00267 (PMC7366733; doi:10.3389/fnhum.2020.00267)
Supplement: Supplementary file 1 [file Data_Sheet_1.docx]

**Supplementary Material**

**Semantic Processing in Autism Spectrum Disorders is Associated with the Timing of Language Acquisition: A Magnetoencephalographic Study**

**Ahtam et al., Frontiers in Human Neuroscience, Speech & Language**

doi: 10.3389/fnhum.2020.00267

1. **Mann-Whitney U Analysis**

We conducted Mann-Whitney U tests to investigate if there are any significant differences between the TD and ASD participants for the responses to the three final word conditions.


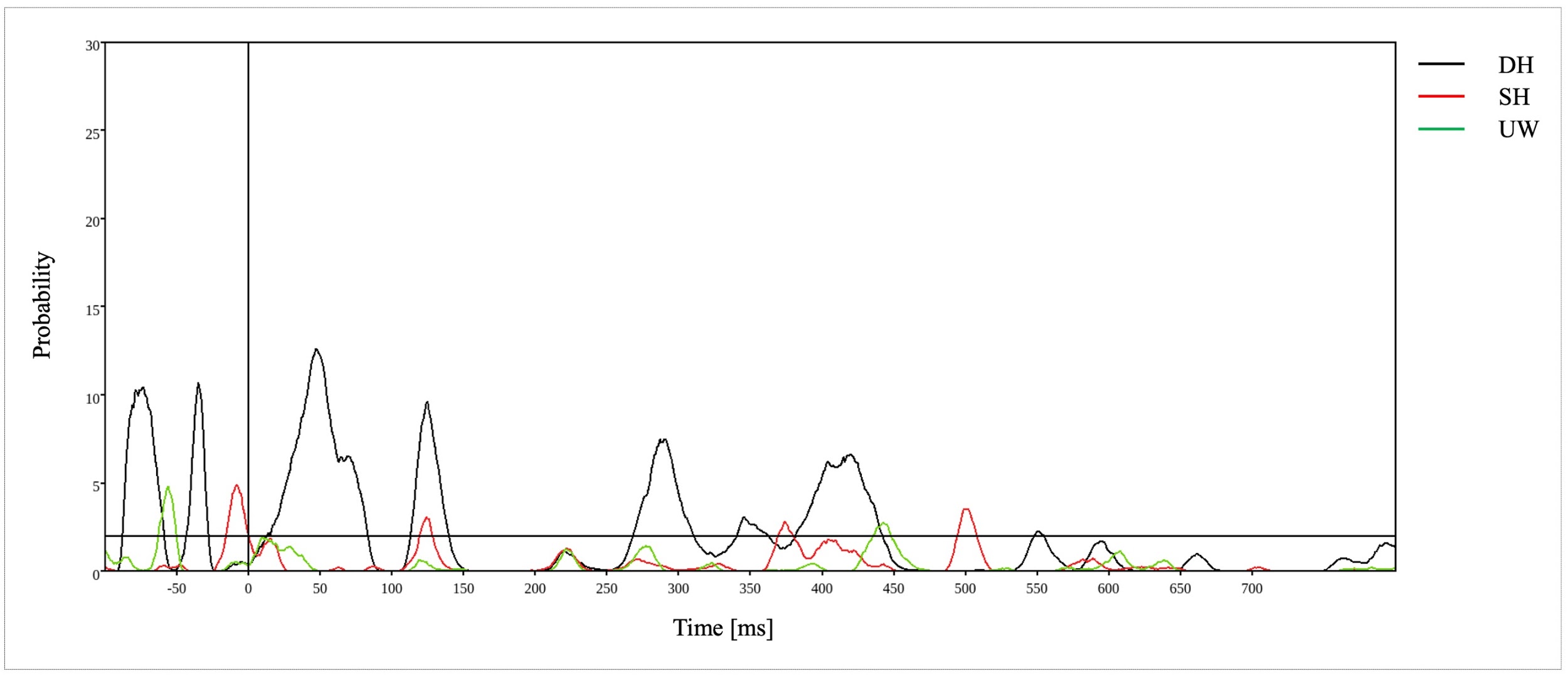


**Supplementary Figure 1A.** Mann-Whitney U test results that show cumulative probability as a function of time for the three final word conditions presenting differences between the TD and ASD participants. Black = dominant homonym, Red = subordinate homonym, Green = unambiguous word. x-axis = time (ms), y-axis = probability where 2 corresponds to *p*=0.01 (solid horizontal line).


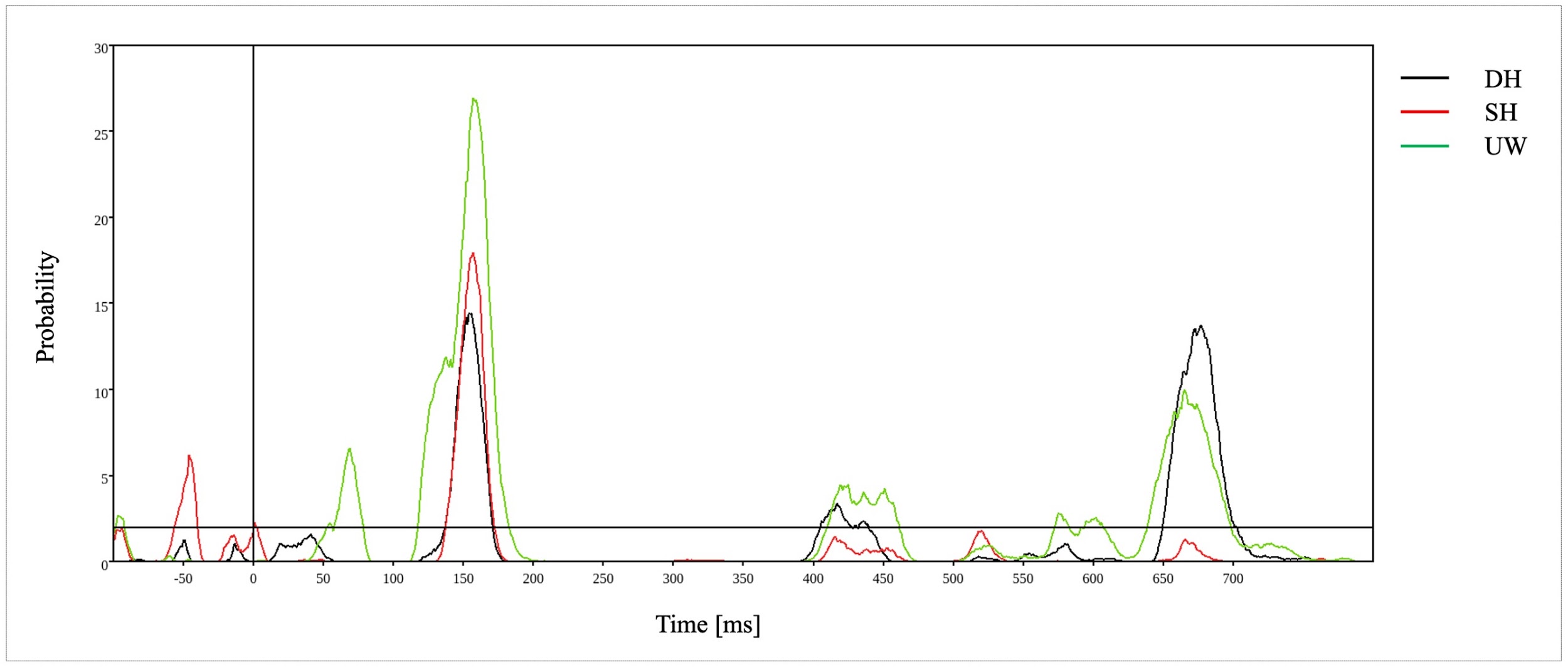


**Supplementary Figure 1B.** Mann-Whitney U test results that show cumulative probability as a function of time for the three final word conditions presenting differences between the NLD and LD participants. Black = dominant homonym, Red = subordinate homonym, Green = unambiguous word. x-axis = time (ms), y-axis = probability where 2 corresponds to *p*=0.01 (solid horizontal line).

The strongest differences between the participant groups between 0-500ms were observed at the early latencies and the N400 latencies. We did not observe any group differences between the LD and NLD at time 350ms. For this reason, we focused our analyses on these two latencies that are related to linguistic processing.

1. **Analysis on Handedness**

We conducted Mann-Whitney U tests to investigate if there are any significant differences between the right-handed LD (N=5) and left-handed LD (N=5) participants for the responses to the three final word conditions.

From the results we can see that at 150ms there is a significant difference between the left-handed LD and right-handed LD groups for the SH and UW final word conditions, but not for the DH final word condition. At the N400 latencies there are no significant differences for any of the final word conditions between the two LD groups.


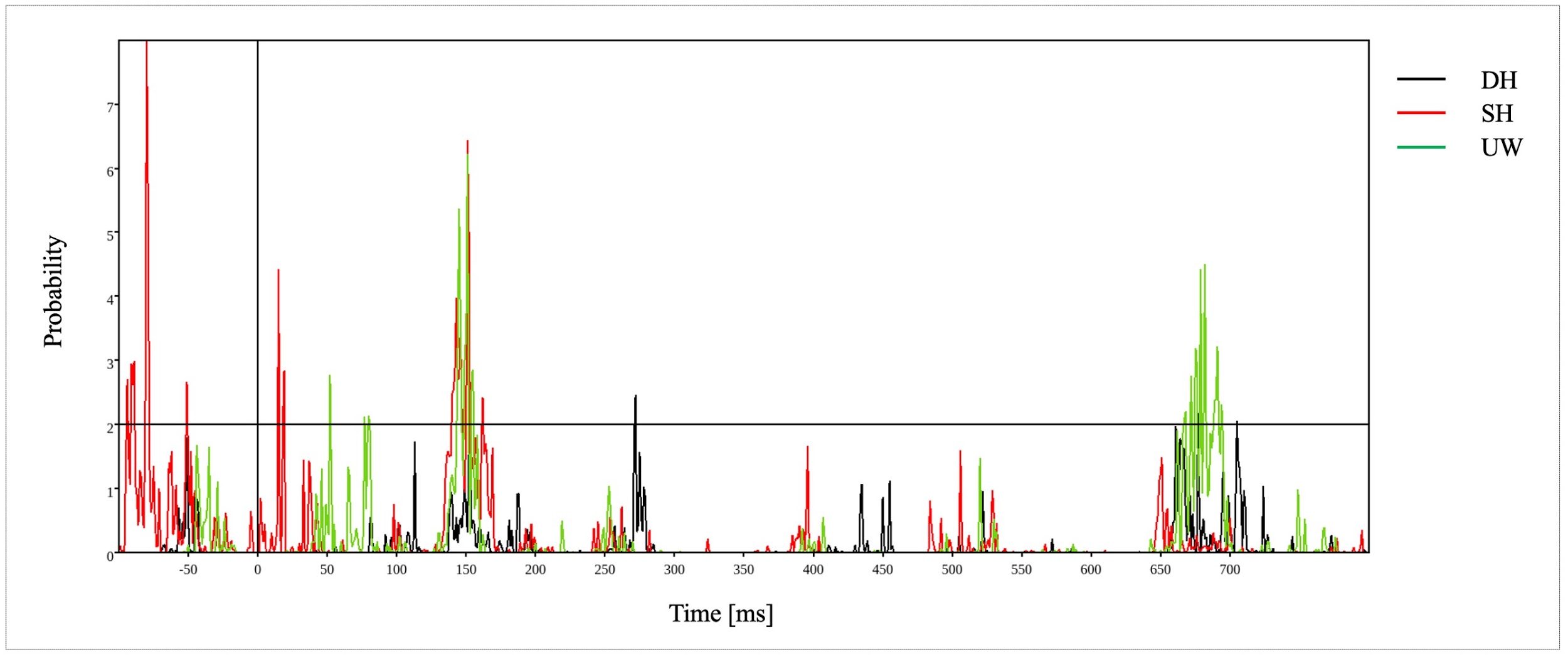


**Supplementary Figure 2A**. Mann-Whitney U test results that show cumulative probability as a function of time for the three final word conditions presenting differences between the right-handed LD and left-handed LD participants. DH = black line, SH = red line, UW = green line. x-axis = time (ms), y-axis = probability where 2 corresponds to *p*=0.01 (solid horizontal line).

**
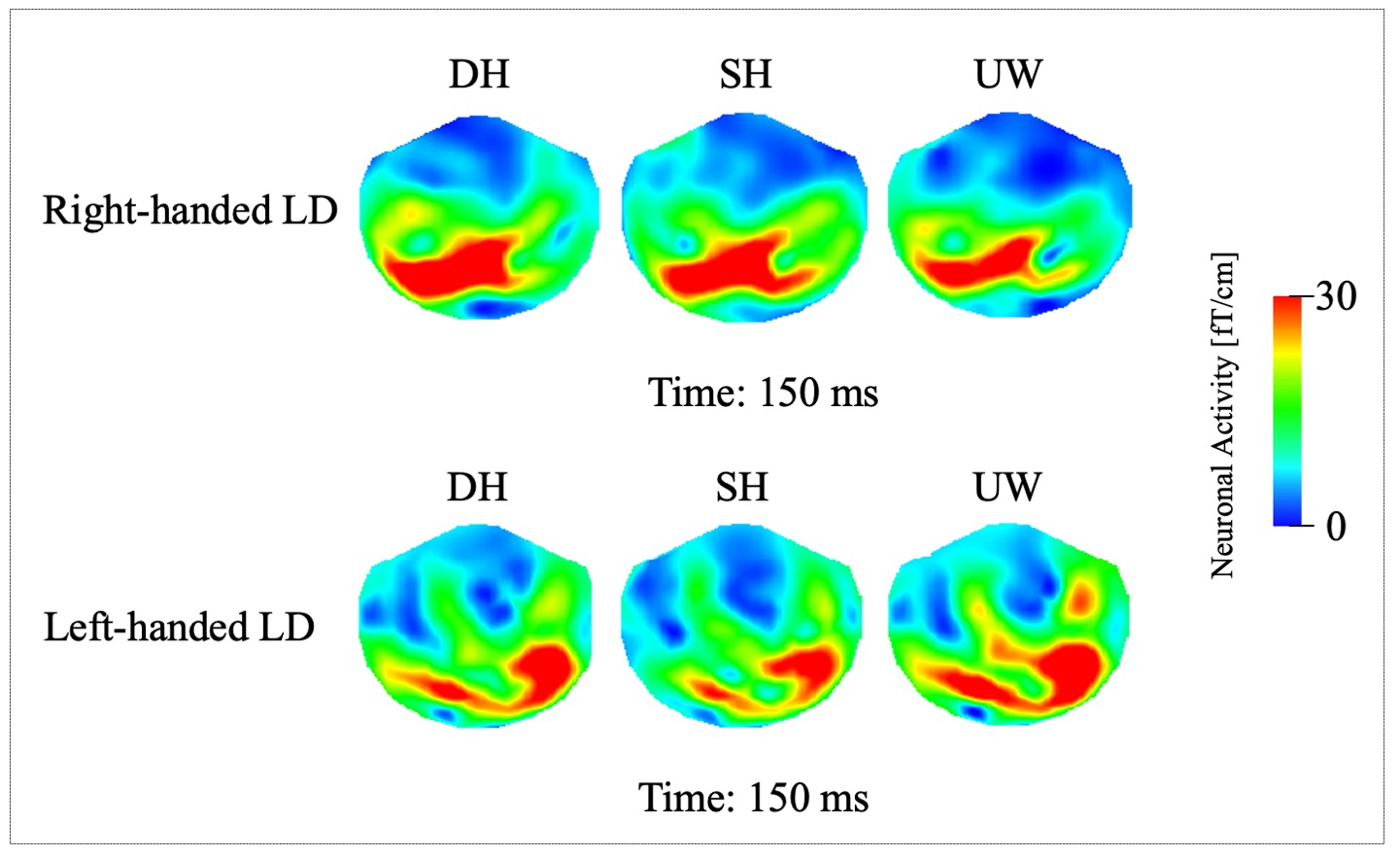
**

**Supplementary Figure 2B.** Local RMS map for the different final word conditions in right-handed LD (N=5) and left-handed LD (N=5) groups at 150ms.

**
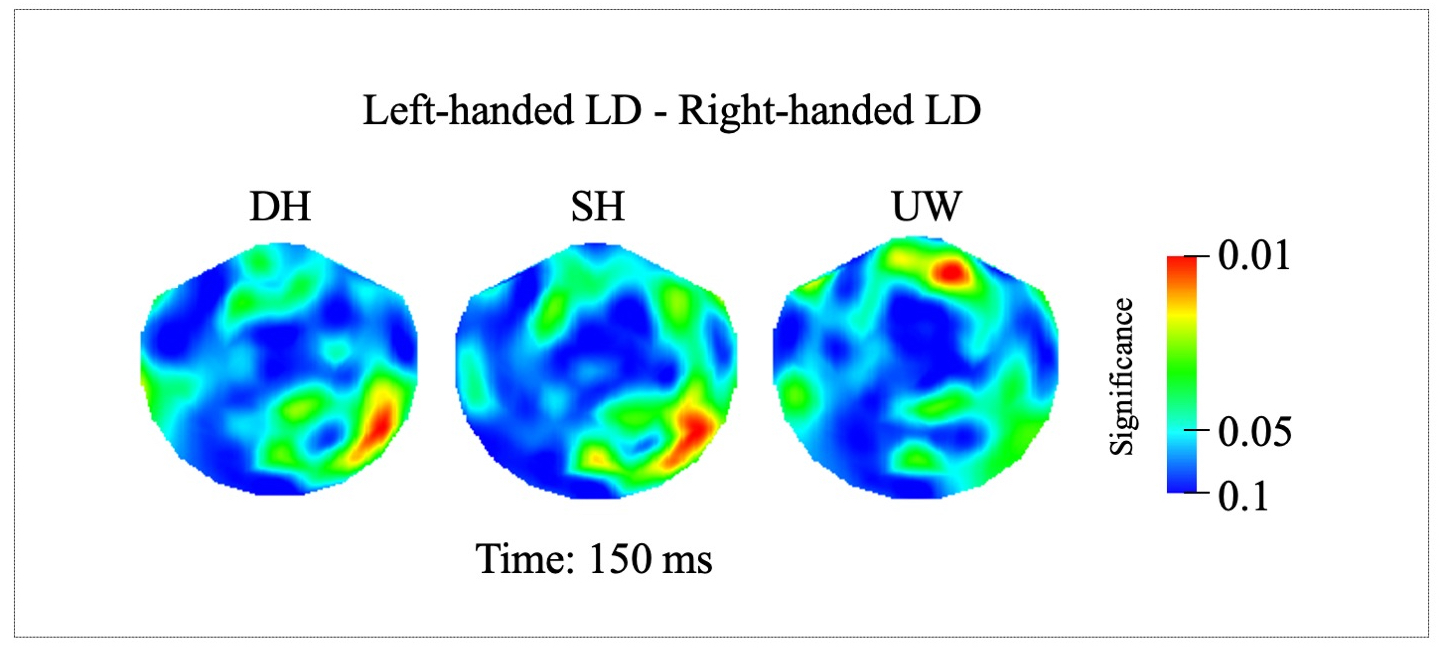
**

**Supplementary Figure 2C.** Mann-Whitney U test results (probability maps) for the three final word conditions presenting the location of significant differences between the right- and left-handed LD participants in the SH and UW conditions (note that the Supp. Figure 1A did not show a significant difference between the right- and left-handed LD groups for the DH condition at 150ms).
